# Gender Differences in Weight Loss Extent Following Bariatric Surgery

**DOI:** 10.3390/jcm14186605

**Published:** 2025-09-19

**Authors:** Santo Colosimo, Federica Sileo, Andrea Gambetti, Francesco Frattini, Amalia Bruno, Sara Mambrini, Luisa Gilardini, Federica Barbera, Alice Gotti, Verdiana Vincenti, Laura Inì, Margherita Novelli, Raffaella Cancello, Gianlorenzo Dionigi, Simona Bertoli

**Affiliations:** 1 Department of Food, Environmental and Nutritional Sciences (DeFENS), University of Milan, 20133 Milan, Italy; 2 Laboratory of Nutrition and Obesity Research, IRCCS Istituto Auxologico Italiano, 20145 Milan, Italyl.gilardini@auxologico.it (L.G.); r.cancello@auxologico.it (R.C.); 3 Bariatric Surgery Unit, IRCCS Istituto Auxologico Italiano, 20145 Milan, Italy; 4 Specialty School of Nutrition Science, University of Milan, 20122 Milan, Italy; 5 Clinical Psychology Service, IRCCS Istituto Auxologico Italiano, 20145 Milan, Italy; 6 Department of Pathophysiology and Transplantation, University of Milan, 20122 Milan, Italy; 7 General Surgery Unit, IRCCS Istituto Auxologico Italiano, 20145 Milan, Italy

**Keywords:** sleeve gastrectomy, gender differences, weight loss

## Abstract

**Background/Objectives:** Metabolic–bariatric surgery (MBS) is a highly effective treatment for severe obesity; however, gender disparities exist in access and postoperative outcomes. Despite men presenting with higher baseline weight and comorbidity burden, they are significantly underrepresented among MBS patients. Existing evidence on gender-specific outcomes remains inconclusive, particularly within Mediterranean populations under updated clinical guidelines. To evaluate gender differences in weight loss outcomes following sleeve gastrectomy in an Italian cohort managed under current multidisciplinary protocols. **Methods:** A prospective observational study was conducted and a total of 131 patients (109 females, 22 males) underwent sleeve gastrectomy at a single center. Anthropometric and metabolic parameters were assessed at baseline and 12 months postoperatively. Outcomes included absolute weight loss (TWL%), percentage of initial and excess body weight lost (EWL%), and waist circumference. Group comparisons used *t*-tests (with Welch’s correction if variances were unequal) and regression models adjusted for baseline BMI and age. **Results:** At 12 months, men achieved significantly greater absolute weight loss than women (−36.6 kg vs. −31.2 kg; *p* = 0.028), although no significant differences were observed for TWL%, EWL%, or waist circumference reduction. Gender remained a significant predictor of absolute weight loss in multivariate analysis, but not of proportional weight loss. Both genders showed similar rates of achieving clinically significant weight loss thresholds. **Conclusions:** While men exhibited greater absolute weight loss, relative weight loss outcomes were comparable between sexes. Gender disparity is observed in the utilization of MBS. These findings highlight the importance of equitable surgical access and tailored postoperative care.

## 1. Introduction

Metabolic and bariatric surgery (MBS) is the most effective long-term intervention for severe obesity, demonstrating consistent outcomes in weight reduction, comorbidity resolution, and improved quality of life [1,2]. Notably, gender differences affect multiple aspects of obesity, including body composition, eating behaviors, obesity phenotypes, and clinical presentations at the time of surgical intervention [3,4,5,6].

Despite similar rates of obesity across sexes, a persistent gender disparity is observed in the utilization of MBS [7,8]. Women consistently represent the majority of surgical candidates, comprising approximately 80% of patients, while men remain underrepresented at around 20% [9]. This imbalance may stem from differences in healthcare-seeking behavior, body image perception, and self-referral patterns [5].

In the current literature, findings regarding gender differences in weight loss outcomes following MBS remain inconsistent [10,11]. Some studies suggest that men tend to lose more absolute weight, while women may achieve greater relative reductions in body mass index or percentage of excess weight lost; however, these patterns are not universally observed and often depend on methodological and population-specific variables [10,11,12]. Despite the global expansion of bariatric research, there is a paucity of recent Italian data, with most national studies dating back almost two decades [11]. To date, no recent studies have specifically investigated whether the introduction in standardized, multidisciplinary protocols has reduced or altered gender differences in surgical outcomes in a modern Italian population.

Contemporary clinical practice emphasizes a more rigorous, multidisciplinary follow-up, in line with evolving international guidelines that stress structured nutritional, psychological, and medical support throughout the postoperative period [13,14,15]. These developments may modulate the relationship between gender and surgical outcomes, making it critical to re-examine gender-related differences in this new clinical context. With this in mind, our study aims to evaluate whether gender-specific differences persist under updated perioperative standards, within an Italian cohort influenced by Mediterranean lifestyle factors. Given the physiological, behavioral, and clinical differences between men and women with obesity, it is imperative to assess whether MBS outcomes are somewhat gender-specific.

## 2. Methods

### 2.1. Patients

Data were prospectively collected from patients attending the Bariatric Surgery Clinic of the IRCCS Istituto Auxologico Italiano (Milan, Italy) as part of a real-life registry of individuals undergoing surgical treatment for obesity. Patient selection followed the SICOB 2023 consensus guidelines [15].

At our center, all patients underwent a comprehensive diagnostic protocol to ensure they met all eligibility criteria for MBS. Once deemed suitable for surgery by the obesity specialist physician, as well as by the registered dietitian and licensed psychologist, patients provided written informed consent and received detailed preoperative instructions including the surgical procedure, diet preparation, and expected postoperative course. Pre-operative education also included lifestyle modification sessions and behavioral strategies to promote long-term success.

Patients were thoroughly educated on the postoperative nutritional protocol, including the gradual reintroduction of food and nutritional supplementation. All patients underwent sleeve gastrectomy and surgery was performed by the same team of surgeons and using the same technique.

After surgery, patients participated in a structured, multidisciplinary follow-up program [16], which included the following.

A surgical consultation two weeks after surgery to monitor healing and address early complications.

Endocrinological and dietetic-nutritional consultations were scheduled at 1, 3, 6, and 12 months postoperatively. Regular counseling by registered dieticians was essential to reinforce healthy eating habits and maintain weight loss. Adequate protein intake with a target of at least 60 g/day was recommended, particularly in the early postoperative period, in order to maintain lean body mass. Lifelong micronutrient supplementation was recommended to prevent common deficiencies such as vitamin B12, vitamin D, iron, and calcium.

Psychological counseling sessions were offered at the same time points (1, 3, 6, and 12 months) to support behavioral change, emotional adjustment, and avoidance of eating disorders.

In addition to scheduled visits, patients had access to on-demand consultations with the multidisciplinary MBS team as needed to receive individualized care and long-term support.

### 2.2. Measures and Statistics

At baseline and during each follow-up visit, data were collected on body weight, height, waist circumference, and blood pressure. Waist circumference was measured at the midpoint between the lower margin of the last palpable rib and the top of the iliac crest, with patients standing and using a non-elastic tape.

Additionally, laboratory assessments included fasting blood glucose and serum creatinine. All data were initially anonymized, implemented on an electronic database (Microsoft Excel^®^), and then exported for analysis using SPSS v.28 (IBM Corp., Armonk, NY, USA).

The following statistical analyses were performed:

Descriptive statistics: Means and standard deviations for continuous variables, frequencies, and 95% confidence intervals for categorical variables.

Group comparisons by gender at baseline using *t*-test or χ^2^-test for independent samples.

Within-group comparisons of anthropometric changes from baseline to 12 months using paired *t*-tests.

Multivariate linear regression was performed to identify independent predictors of absolute weight loss and percentage initial body weight loss (TWL%) at 12 months, adjusting for sex, age, and baseline BMI.

Categorical analyses (e.g., achieving ≥30% TWL% or ≥50% percentage excess body weight loss (EWL%)) were performed using the χ^2^-test.

Normality of distributions (Shapiro–Wilk) and homogeneity of variances (Levene’s) were assessed; when heteroscedasticity was detected, Welch’s *t*-test was applied.

Missing data were minimal (<5%) and handled using complete case analysis (listwise deletion).

A two-sided *p*-value < 0.05 was considered statistically significant for all comparisons.

### 2.3. Sample Size and Power Considerations

A post hoc power analysis was conducted based on the observed difference in absolute weight loss between men and women at 12 months post-surgery (mean difference = 5.4 kg, SD ≈ 10.3 kg, Cohen’s *d* = 0.52). With a total sample of 131 patients (109 females and 22 males), the achieved power to detect a between-group difference at α = 0.05 was approximately 76–79%. Although slightly under the conventional 80% threshold, this study successfully identified a statistically significant gender difference in absolute weight loss. For a balanced design (1:1 allocation), a total sample of approximately 74 participants (37 per group) would have been sufficient to detect a similar effect size with 80% power.

### 2.4. Ethics

This study was conducted in accordance with the ethical principles set forth in the Declaration of Helsinki. The research protocol received prior approval from the Ethical Committee of the Istituto Auxologico Italiano (Milan, Italy), and all procedures complied with applicable regulatory requirements. Written informed consent was obtained from all participants after providing a detailed explanation of this study’s objectives, methods, potential risks, and anticipated benefits.

## 3. Results

### 3.1. Baseline Characteristics of the Population

The cohort included 131 patients undergoing sleeve gastrectomy, including 109 females (83.2%) and 22 males (16.8%) (Table 1). The mean age at baseline was 45.5 years (SD = 10.6), with no significant age difference between genders (*p* = 0.435). Males presented with significantly higher baseline body weight compared to females (134.8 ± 16.7 kg vs. 109.4 ± 14.8 kg, *p* < 0.001), although BMI values were comparable (42.3 ± 4.0 vs. 41.8 ± 4.2, *p* = 0.605). Baseline waist circumference was significantly higher in males (134.3 ± 9.7 cm) than in females (118.9 ± 11.0 cm, *p* < 0.001). The prevalence of type 2 diabetes was not significantly different between genders (12.1% in females vs. 4.8% in males, *p* = 0.321), nor was the prevalence of hypertension, (59.1% vs. 38.7%, *p* = 0.078). Fasting blood glucose levels tended to be higher in males (113.8 ± 56.7 mg/dL) than in females (100.5 ± 18.1 mg/dL), though this did not reach statistical significance (*p* = 0.404).

### 3.2. Weight Loss Outcomes

Paired *t*-tests revealed a significant reduction in body weight over time across the entire sample (−34.3 kg ± 1.3 kg average difference *p* < 0.001).

**Figure 1:**
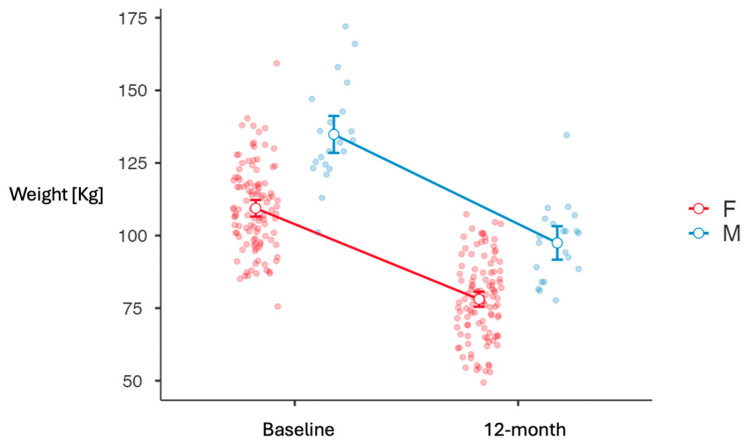
Absolute 12-month weight change [kg] following sleeve gastrectomy. F, female; M, male.

**Figure 2:**
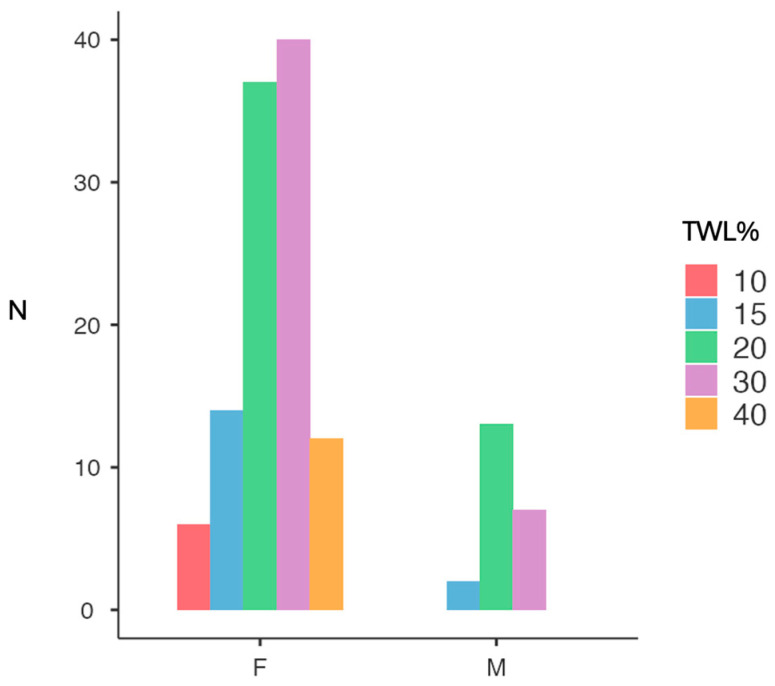
TWL% brackets across genders. F, female; M, male; TWL%, percentage total weight loss.

Independent sample *t*-tests highlighted that males experienced a significantly greater mean weight change at 12 months (weight change = −36.6 kg ± 10.3) compared to females (weight change = −31.2 kg ± 10.4) (Figure 1), with a mean difference of 5.39 kg (*p* = 0.028, Cohen’s *d* = 0.52) (Table 2). In contrast, no statistically significant gender differences were found for the percentage of TWL% (−27.2 vs. −28.6, females vs. males, respectively, *p* = 0.481) although females had a slightly higher mean. When stratified by percentage of weight loss from baseline, 38.2% of all patients lost approximately 20% of their initial weight, 35.9% achieved a 30% reduction, and 9.2% reached or exceeded a 40% loss (*p* < 0.01) (Figure 2). Gender-specific patterns revealed that women tended to distribute more evenly across weight loss brackets, with 33.9% in the 20% category, 36.7% in the 30% category, and 11.0% reaching 40% or more. Conversely, male patients were more concentrated in the 20% loss group (59.1%), with fewer achieving 30% loss (31.8%) and none reaching the 40% threshold, but these proportions were not significantly different from those of the female population (χ^2^ = 7.00, df = 4, *p* = 0.136).

**Figure 3:**
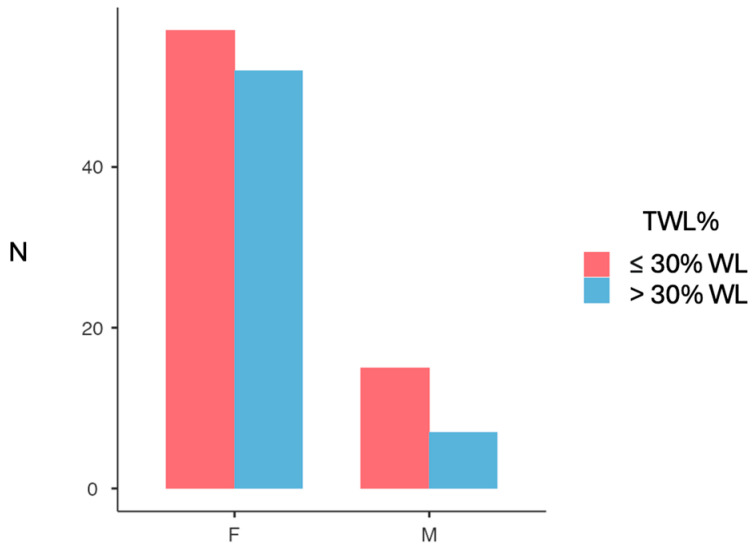
Participants achieving 30% total weight loss (TWL%) following sleeve gastrectomy, split by gender. F, female; M, male.

In terms of the ≥30% weight loss threshold, 45.0% of all patients achieved this threshold. Although a higher absolute proportion of women reached this goal (47.7% vs. 31.8%), the difference between sexes did not reach statistical significance (χ^2^ = 1.87, *p* = 0.172) (Figure 3).

Likewise, no significant changes emerged from the EWL% comparison (48.4 ± 15.3 in females and 45.9 ± 12.05 in males, *p* = 0.46) (Table 2).

### 3.3. Linear Regression Analyses

Multivariate linear regression for absolute total weight change indicated that age (*p* < 0.001), gender (*p* = 0.008), and baseline BMI (*p* = 0.017) were significant predictors (Table 3). Males were predicted to lose an additional 5.89 kg compared to females (β = −5.89, SE = 2.20) after adjusting for age and BMI (Table 3). However, in models predicting TWL%, only age remained a significant predictor (*p* < 0.001); gender was not significant (β = 0.85, *p* = 0.653) (Table 4). When smoking and diabetes status were included, age retained its predictive value (*p* < 0.001), while gender again showed no significant effect.

### 3.4. Waist Circumference

Analysis of the change in WC data revealed no significant gender differences (Student’s *t* = 0.748, *p* = 0.456). The mean change was slightly greater in males (−30.2 ± 7.81) than in females (−28.0 ± 10.6), but this difference was not statistically or clinically significant (Table 2).

## 4. Discussion

Our study confirms that sleeve gastrectomy is a highly effective intervention for substantial weight loss and waist circumference reduction in both men and women. While men exhibited a greater absolute weight loss at 12 months compared to women (mean difference of 5.39 kg, *p* = 0.028), no significant gender difference was observed in relative measures such as IBW%L or EWL%. These results suggest that although men tend to lose more in absolute terms—likely due to their higher initial body weight—both genders derive comparable relative benefits from surgery. These findings align with earlier reports that men typically lose more kilograms post-surgery, whereas women may have similar or better proportional outcomes [10,11].

Importantly, our regression analyses revealed that gender remained an independent predictor of absolute weight loss, even after adjusting for age and baseline BMI, but not for TWL%. This supports the hypothesis that gender-associated physiological factors, such as body composition and metabolic rate, may partially explain differences in weight loss magnitude [5,6]. Notably, baseline BMI and age were also significant predictors, underscoring the multifactorial nature of surgical weight loss.

Our results also show that gender-specific differences in absolute weight loss do not lead to differences in waist circumference reduction. Both genders achieved comparable improvements in central obesity after 12 months. This suggests that the visceral fat component—which is closely linked to cardiometabolic risk—responds similarly to surgery in men and women, supporting the metabolic efficacy of sleeve gastrectomy in both sexes.

Our cohort’s gender distribution (83% women, 17% men) mirrors global patterns and reinforces the persistent gender disparity in access to MBS [9]. This trend is concerning given that men in our study, consistent with previous reports, presented with significantly higher baseline weight and waist circumference, and tended to have worse metabolic profiles, including higher rates of hypertension and elevated fasting glucose, although these were not statistically significant [17]. These differences likely reflect a delayed referral of male patients, as supported by other studies showing that men often undergo surgery later in the disease course, with a heavier comorbidity burden [12,17]. Nevertheless, given the relatively small number of male patients, caution is required when generalizing our findings, and the results should be interpreted with this limitation in mind.

Behavioral and psychological dimensions may further explain outcome patterns. Women are more prone to emotional eating—a maladaptive coping mechanism involving food consumption in response to negative emotions—whereas men are more likely to engage in external eating, driven by environmental cues [18,19]. Emotional eating is associated with higher caloric intake and less consistent adherence to dietary guidance, particularly under stress, which could potentially affect the pace and consistency of weight loss [20]. However, our standardized, multidisciplinary postoperative care—including counseling and dietary support—may have mitigated these behavioral barriers, leading to comparable long-term outcomes. Behavioral mechanisms such as emotional eating in women or external eating in men were discussed to provide context; however, we acknowledge that these interpretations are speculative, as such data were not collected in the present study.

Interestingly, despite gender-based baseline differences in weight and waist circumference, both groups demonstrated similar reductions in waist circumference at 12 months. This suggests that visceral fat loss—strongly associated with metabolic improvements—was not significantly different by sex, supporting the metabolic efficacy of sleeve gastrectomy in both men and women.

A key strength of this study lies in its rigorous protocol, reflecting updated guidelines by both international and national societies [15,16,21,22]. Frequent contact with the nutritional team allowed the patients to adhere to a structured dietary progression—from clear liquids to solid foods—over several weeks to prevent complications like vomiting and other gastrointestinal symptoms. Also, the continuous medical and psychological support granted increased adherence to postoperative instructions, fostered weight loss pace, and prevented weight regain.

## 5. Limitations

Nevertheless, some limitations should be acknowledged. The most important limitation is the gender imbalance, with men strongly underrepresented compared to women. This reduces the statistical power for subgroup analyses and may overestimate or underestimate true gender effects. The relatively small sample size, particularly for male patients, limits statistical power for detecting gender differences in categorical weight loss brackets and may underestimate subtle behavioral or metabolic interactions. Another limitation is the potential referral bias: men often undergo bariatric surgery later than women, typically with a higher baseline weight and heavier metabolic burden. This may have influenced both baseline characteristics and observed outcomes in our cohort. Additionally, the absence of psychological questionnaire data or measures of dietary adherence prevents a deeper exploration of how gender-specific behaviors influenced the outcomes. Future studies should include behavioral assessments and longer follow-up periods to explore differences in weight maintenance and metabolic remission to elucidate the complex interplay of gender in bariatric outcomes. Such insights will inform tailored interventions that optimize weight loss and health improvements for all patients.

## 6. Conclusions

Our findings confirm that both men and women benefit substantially from sleeve gastrectomy under the most recent clinical protocols.

While men tend to lose more weight in absolute terms, gender does not appear to significantly affect relative weight loss outcomes or central adiposity reduction.

These results highlight the importance of early equitable access to MBS across genders, alongside personalized, multidisciplinary management to support optimal outcomes.

## Figures and Tables

**Table 1:** Baseline characteristic comparison of 131 patients living with obesity and undergoing sleeve gastrectomy. Mean values followed by standard deviation in brackets. BMI, body mass index. T2D, type 2 diabetes mellitus.

	Female (SD) *n* = 109	Male (SD) *n* = 22	Total (SD) *n* = 131	*p* Value
Age [years]	45.2 (10.8)	47.1 (9.8)	45.5 (10.6)	0.435 ^1^
Weight [kg]	109.4 (14.8)	134.8 (16.7)	113.7 (17.8)	<0.001 ^1^
BMI [kg/m^2^]	41.8 (4.2)	42.3 (4.0)	41.9 (4.2)	0.605 ^1^
Waist [cm]	118.9 (11.0)	134.3 (9.7)	121.6 (12.2)	0.001 ^1^
Glycaemia [mg/dL]	100.5 (18.1)	113.8 (56.7)	102.6 (28.4)	0.404 ^3^
T2D [%]	12.1	5	11	0.321 ^2^
Hypertension [%]	39	59	42	0.078 ^2^

^1^ Independent *t*-test. ^2^ χ^2^ test. ^3^ Welch’s *t*-test.

**Table 2:** Twelve-month postoperative outcomes of 131 sleeve gastrectomy patients. Changes in body weight. Mean values followed by standard deviation in brackets. TWL% 12M, percentage total weight loss after 12 months. EWL% 12M, percentage excess weight loss after 12 months.

	Female (SD) *n* = 109	Male (SD) *n* = 22	*p* Value
Weight Loss 12M [kg]	−31.2 (10.40)	−36.6 (10.33)	0.028 ^1^
TWL% 12M	−28.6 (8.99)	−27.2 (6.24)	0.48 ^2^
EWL% 12M	48.4 (15.25)	45.9 (12.05)	0.46 ^2^
Waist Circumference (cm)	−28.0 (10.6)	−30.2 (7.81)	0.45 ^1^

^1^ Independent *t*-test. ^2^ χ^2^ test.

**Table 3:** Regression analysis for 12-month body weight loss and gender, adjusted for age and baseline BMI. r = 0.479, r^2^ = 0.229. BMI, body mass index.

Predictor	Estimate (β)	SE	95% Confidence Interval		*p* Value
			Lower	Upper	
Gender M-F	−5.89	2.197	−10.233	−1.537	0.008
Age	0.379	0.078	0.225	0.543	<0.001
BMI	−0.479	0.197	−0.868	−0.089	0.017

**Table 4:** Regression analysis for 12-month TWL% and gender, adjusted for age. r = 0.368, r^2^ = 0.135. M: male; F: female.

Predictor	Estimate (β)	SE	95% Confidence Interval		*p* Value
			Lower	Upper	
Gender M-F	0.850	1.886	−2.881	4.582	0.653
Age	0.293	0.067	0.162	0.425	<0.001

## Data Availability

Dataset available on request from the authors.
